# Plasma Protein Panel for Assessing the Risk of Alzheimer’s Disease by MRM-MS Analysis: The Study of Two Independent Clinical Cohorts

**DOI:** 10.3390/ijms27010015

**Published:** 2025-12-19

**Authors:** Polina A. Strelnikova, Alexey S. Kononikhin, Natalia V. Zakharova, Anna E. Bugrova, Maria I. Indeykina, Yana B. Fedorova, Igor V. Kolykhalov, Anna Y. Morozova, Alisa V. Andryushchenko, Elena D. Fedoseeva, Marina A. Emelyanova, Dmitry A. Gryadunov, Svetlana I. Gavrilova, Vladimir A. Mitkevich, George P. Kostyuk, Yulia A. Chaika, Alexander A. Makarov, Evgeny N. Nikolaev

**Affiliations:** 1 Project Center of Omics Technologies and Advanced Mass Spectrometry, 121205 Moscow, Russia; pauline.strel@gmail.com (P.A.S.); nvzakharova@yandex.ru (N.V.Z.); anna.bugrova@gmail.com (A.E.B.); mariind@yandex.ru (M.I.I.); 2 Emanuel Institute for Biochemical Physics, Russian Academy of Sciences, 119334 Moscow, Russia; 3 Mental Health Research Center, 115522 Moscow, Russia; yfedorova@yandex.ru (Y.B.F.); ikolykhalov@yandex.ru (I.V.K.); sigavrilova@yandex.ru (S.I.G.); berseneva76@yandex.ru (Y.A.C.); 4 Russian Medical Academy of Postgraduate Education, Ministry of Healthcare of the Russian Federation, 125993 Moscow, Russia; 5 Mental-Health Clinic No. 1 Named After N.A. Alexeev of Moscow Healthcare Department, 117152 Moscow, Russia; hakurate77@gmail.com (A.Y.M.); alissia.va@mail.ru (A.V.A.); kgr@yandex.ru (G.P.K.); 6 Department of Mental Health, Faculty of Psychology, M. V. Lomonosov Moscow State University, 119991 Moscow, Russia; 7 Engelhardt Institute of Molecular Biology, Russian Academy of Sciences, 119991 Moscow, Russia; elfed0@mail.ru (E.D.F.); emel_marina@mail.ru (M.A.E.); gryadunov@gmail.com (D.A.G.); mitkevich@gmail.com (V.A.M.); aamakarov@eimb.ru (A.A.M.); 8 Department of Psychiatry, Psychotherapy and Psychosomatics, Sechenov First Moscow State Medical University, Ministry of Health of the Russian Federation, 119435 Moscow, Russia

**Keywords:** Alzheimer’s disease, mild cognitive impairment, proteomics, biomarkers, blood, proteins, mass-spectrometry

## Abstract

Early recognition of a risk of Alzheimer’s disease (AD) remains a global challenge, and blood proteomic markers are of particular interest for wide-scale diagnostic use. Quantitative multiple reaction monitoring (MRM) approach demonstrates good reproducibility in the characteristic changes in the levels of reported candidate biomarkers (CBs) in different cohorts in AD. Following up on our previous study, we performed a joint analysis of 331 blood plasma samples from two different clinical cohorts of participants, comprising a total of 95 samples from patients with AD, 136 samples from patients with mild cognitive impairment (MCI), and 100 samples from controls. The obtained results confirm the significance of 37 CBs. A logistic regression-based algorithm was used to build protein classifiers, and a total of 21 important proteins were selected, 13 of which (ORM1, APOA4, LBP, HP, FN1, BCHE, APOE, PZP, A1BG, TF, SERPINA7, TTR, and F12) formed a universal panel that demonstrated strong classification performance in distinguishing AD patients from controls (ROC-AUC = 0.90) and in separating stable and progressing patients with MCI (ROC-AUC = 0.81). Overall, the analysis confirms the high potential of the MRM method for validating CBs in independent cohorts.

## 1. Introduction

Alzheimer’s disease (AD) is a progressive neurodegenerative disorder characterized by cognitive decline and significant socioeconomic impact. Although neuroimaging techniques and cerebrospinal fluid (CSF) biomarkers have transformed research and diagnostics, their invasive nature, high costs, and limited accessibility restrict widespread implementation [1]. Consequently, there is an urgent need for minimally invasive, scalable, and cost-effective biomarkers. Among new approaches, blood-based biomarkers are attracting the most attention, as they combine economic feasibility with patient comfort due to the minimally invasive nature of blood sampling, while holding strong potential for successful integration into routine clinical practice and large-scale screening initiatives.

In recent years, significant progress has been made in plasma assays targeting key AD biomarkers such as amyloid-β (Aβ42/40) and phosphorylated tau (p-tau181, p-tau217), which are increasingly recognized as reliable correlates of pathology previously measured only in CSF [2,3]. According to recent evidence, elevated levels of p-tau181 and p-tau217—as well as their combination with NfL or GFAP—have shown strong prognostic power for predicting the risk of AD up to ten years before clinical onset [4]. In 2025, the FDA approved two landmark blood-based assays for AD. The first, Lumipulse G pTau217/β-Amyloid 1-42 Plasma Ratio (Fujirebio Diagnostics), was cleared in May 2025 and demonstrated high accuracy in detecting amyloid pathology [5,6,7]. The second, Elecsys pTau181 Test (Roche Diagnostics/Eli Lilly), approved in October 2025, is the only test intended to aid clinicians in diagnosing AD in patients with cognitive symptoms [8]. These advances represent a major leap forward in AD diagnostics, bringing closer the possibility of widespread implementation of early diagnosis at the population level. However, experts note that these tests do not replace clinical assessment and should only be used as part of a comprehensive diagnostic evaluation [9].

In parallel with the development of test systems based on “core” biomarkers, active work is underway to study the proteome of biological fluids using multiplex methods. For example, the recently launched Global Neurodegeneration Proteomics Consortium aggregates large-scale proteomic datasets from plasma, serum, and CSF studies across various neurodegenerative disorders, including AD [10]. The rationale for investigating the full proteome of biological fluids, rather than focusing solely on individual biomarkers, is supported by several key considerations. Population-based data suggest that the proportion of patients with “mixed type” dementia may be 50% or more [11]. Moreover, AD frequently co-occurs with systemic conditions, the most significant of which are cardiovascular pathologies, as well as diabetes [12,13]. Such factors have a profound impact on the proteome of biological fluids. Changes in plasma protein composition may reveal biologically distinct forms of AD, consistent with recent CSF proteomic findings defining five molecular subtypes with unique genetic risk profiles [14]. At the same time, these studies can improve our understanding of the molecular mechanisms driving the pathology and point to new therapeutic targets.

The main multiplex platforms used in large-scale proteomics today include Olink^®^ Proximity Extension Assay (PEA) technology [15], SomaScan^®^ aptamer-based proteomics [16], and various mass spectrometry-based (MS) approaches. High-throughput affinity-based approaches such as Olink^®^ and SomaScan^®^ provide scalable, reproducible quantification suited to large-scale studies; however, their mutual comparability—and alignment with mass-spectrometry data—still requires careful evaluation, as cross-platform correlations vary substantially [17,18,19]. MS remains “the gold standard” for proteomic discovery and validation, offering unbiased detection of protein signals. Targeted MS methods such as MRM and PRM are now widely used to verify and quantify biomarkers found with large-scale affinity platforms. While multiplexed MS typically quantifies fewer proteins than affinity-based assays (hundreds rather than thousands), it brings key advantages to biomarker research: high specificity, strong reproducibility, and independence from antibody reagents [20,21]. In particular, the BAK 125 kit developed by MRM Proteomics Inc. (Montreal, QC, Canada) illustrates a well-established implementation of this methodology. Validated under Clinical Proteomic Tumor Analysis Consortium (CPTAC) guidelines, the kit quantifies more than 100 plasma proteins using as little as 10 µL of sample [22,23,24].

In the context of AD, the number of plasma protein biomarkers reproducible in different cohorts that may have diagnostic or prognostic potential has already exceeded a hundred, and 62 potential AD markers were reproduced in three or more independent cohort studies [25]. More recent use of Olink^®^ PEA has revealed a much broader set of biomarker candidates compared to earlier studies employing mass spectrometry or conventional multiplex immunoassays (e.g., ELISA, Luminex xMAP) [25,26,27,28]. This likely reflects its improved sensitivity for detecting low-abundance proteins—particularly those related to inflammatory signaling, which plays a critical role in AD pathogenesis [29,30]. Nevertheless, the most consistently replicated biomarkers (reported in more than five cohorts) include PPY, A2M, APOE, C3, IGFBP2, FGG, and APOA4 [25].

To continue targeted proteomic studies and follow up on our previous MRM-MS study [31], comparison of results obtained in different patient cohorts appears to be particularly important for further confirmation of the significance of individual potential markers. The chosen BAK 125 proteins panel included 75 proteins previously identified as potential biomarkers of AD [31]. Therefore, the primary objective of the current study was to perform an MRM-based analysis with the BAK125 assay for non-depleted plasma samples obtained from participants who were recruited in two clinical centers (Moscow, Russia), including patients with AD, mild cognitive impairment (MCI), and age-matched controls (in total *n* = 331). Most participants had been followed longitudinally, allowing the identification of a subgroup of MCI patients who exhibited clinical progression over time.

As a result, we developed a universal protein panel that demonstrated strong classification performance in distinguishing AD patients from controls, as well as in separating stable patients with MCI from progressing to AD.

## 2. Results

### 2.1. Combining Two Clinical Cohorts and MRM Proteomic Analysis

The study included samples obtained from two clinical centers (see Section 4): N.A. Alekseev Mental-Health Clinic No. 1 (MH1) and Mental Health Research Center (MHRC). The MH1 cohort comprises 49 plasma samples from patients diagnosed with Alzheimer’s disease (AD), 78 from individuals with mild cognitive impairment (MCI), and 49 from cognitively unimpaired controls. The MHRC cohort was based on a previously described [31] but was expanded to include plasma samples from 46 patients with AD, 58 from individuals with MCI, and 51 from controls. Among the battery of cognitive assessments administered across clinics, only the MMSE and CDT were common to both (see Section 4).

The MRM analysis of absolute concentrations of 125 plasma proteins identified 82 proteins that were consistently quantified in both cohorts (Supplementary Table S1). These proteins (a) met all technical MRM assay quality criteria (see Section 4), (b) showed intra-sample variability below 20% in repeated measurements, and (c) were detected in more than 70% of samples.

In the MH1 cohort, 28 proteins reached statistical significance (*q*-value < 0.05) in the AD vs. Control comparison, whereas only five proteins (AFM, PON1, FGB, FN1, FGG) were significant in the MHRC cohort (Supplementary Table S2). This finding is consistent with our earlier analysis on a smaller dataset from the same clinical center, where only AFM passed the 5% FDR cutoff, whereas PON1 passed the 10% cutoff together with APOE [31]. Notably, the latter exhibited an adjusted *p*-value of approximately 0.09 in the current analysis.

**Figure 1:**
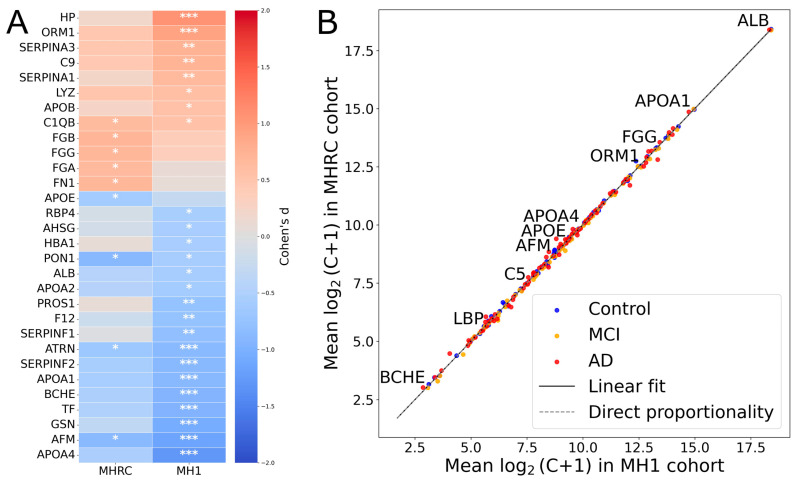
The comparison of MRM results across MHRC and MH1 cohorts. (**A**) The top 30 proteins with the largest absolute effect sizes (|Cohen’s *d*|) in either center (MH1 or MHRC). Asterisks indicate statistical significance: *** for *q* < 0.001, ** for *q* < 0.01, * for *q* < 0.1. (**B**) Comparison of mean protein concentrations for AD/MCI/Control groups across two clinical centers.

For comparison between centers, the top 30 proteins showing the highest absolute effect sizes (|Cohen’s *d*|) in at least one cohort (MH1 or MHRC) were selected for visualization (Figure 1A). It is noteworthy that 15 proteins showed opposite changes in level regulation in the two cohorts (PROS1, HBA1, C2, PRDX2, CA1, CPB2, HPX, SERPIND1, APOD, ADIPOQ, IGHM, CLU, C1RL, FBLN1, CD5L) (Supplementary Table S2). None of them were significant in comparison between the Control and AD groups in the MHRC cohort (*q*-value > 0.5). Only PROS1, HBA1, C2, PRDX2, and CA1 among the mentioned proteins had a *q*-value < 0.1. Thus, overall, the opposite changes can be considered negligible, as for the majority of proteins, the observed differences in concentrations appear to be random according to their *p*-values. The high correlation coefficient (R ≈ 0.99) between group-averaged protein concentrations (log_2_ (C + 1) scale) further supports the consistency of the data between the two clinical cohorts (Figure 1B).

**Figure 2:**
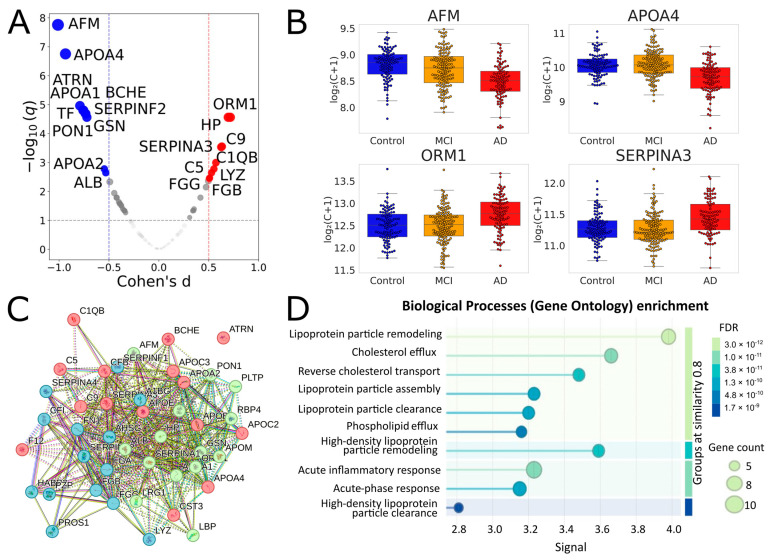
Identification and functional analysis of significantly different proteins for comparison of Controls vs. AD in a joint analysis of MHRC and MH1 cohorts. (**A**) Volcano plot for the AD vs. Control comparison in the combined cohort. Proteins with |Cohen’s *d*| > 0.5 and *q* < 0.1 are highlighted. Red indicates proteins upregulated in AD, and blue indicates proteins downregulated. (**B**) Selected boxplots for significant proteins. (**C**) Results of the STRING k-means clustering (https://string-db.org) of 43 significantly different proteins between AD and Control groups (listed in Table 1) with default network-parameters settings (network type—full STRING network, all active interaction sources, minimum required interaction score—medium confidence); red—proteins involved in the chylomicron remodeling and cholesterol efflux; green—blood microparticle, complement and coagulation cascades; blue—platelet degranulation and fibrinolysis. (**D**) Biological processes (Gene Ontology) enrichment for the 43 significantly different proteins.

In the combined dataset, 41 proteins showed adjusted *q* < 0.05, and 43 proteins passed the 10% FDR cutoff (Table 1, Figure 2, Supplementary Table S3). Of the 28 proteins that were significant in the MH1 cohort, 26 overlapped with this set. All significant proteins of the MHRC cohort were also in this set. It seems especially important to note that 19 of the 43 proteins were selected for the binary classification in our previous study on the MHRC cohort; therefore, the result on the current joint analysis reinforces their significance. Importantly, the performed STRING analysis (Figure 2C,D) revealed very close relationships between the 43 significantly different proteins and their essential involvement in interconnected biological processes (including inflammation, fibrinolysis, complement, and coagulation cascades), the changes in which are characteristic of AD.

When comparing the Control and MCI groups, six proteins (SERPINF2, CFI, TF, FN1, PON1, CFB) reached a *q* ~ 0.20 (Supplementary Table S4). Three of them (except CFI, FN1, and CFB) were among the top 10 most significant proteins in the Control vs. AD comparison, while CFI, FN1, and CFB also demonstrated *q* < 0.05.

### 2.2. Developing of Universal Protein Panel for Assessment of the RISK of Progression to AD

**Figure 3:**
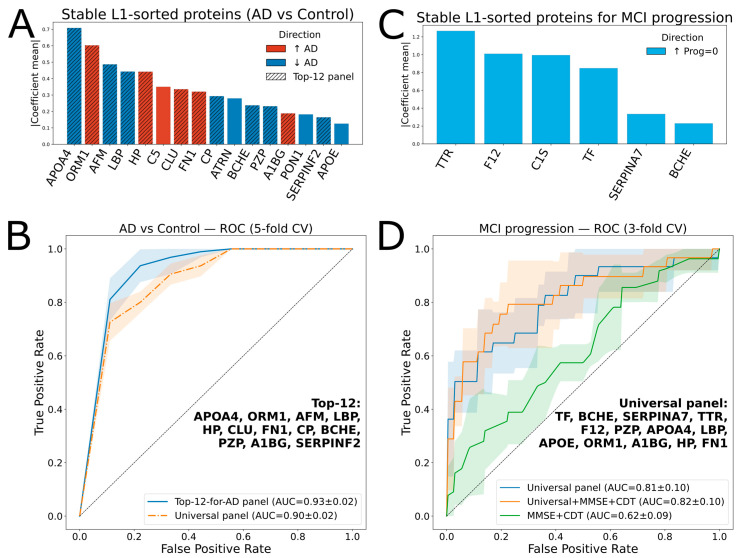
Protein ranking and ROC analysis for classifications of AD vs. Control, and progressed MCI vs. stable MCI. (**A**) Ranked proteins for AD vs. Control classification; the hatched bars indicate 12 proteins (Top-12 panel) selected for the AD vs. Control classifier. (**B**) ROC curves for AD vs. Control classification with the Top-12 and universal panels, using 5-fold cross-validation with a logistic regression model. Shaded areas indicate variability across cross-validation folds, while the gray dashed line represents chance-level performance (AUC = 0.5). (**C**) Ranked proteins for MCI vs. stable MCI classification. (**D**) ROC curves for classification using 3-fold cross-validation with a logistic regression model with different feature sets. Shaded areas indicate variability across cross-validation folds, while the gray dashed line represents chance-level performance (AUC = 0.5).

A linear regression-based algorithm was used as the core of the AD vs. Control classification model. Sixteen proteins (APOA4, ORM1, AFM, LBP, HP, C5, CLU, FN1, CP, ATRN, BCHE, PZP, A1BG, PON1, SERPINF2, and APOE) were selected in 100% of the runs for L1-regularization with non-zero coefficients (referred to as “stable”) (Figure 3A). Most of them (except CLU and CP) showed statistical significance (Table 1). The best ROC-AUC of 0.93 ± 0.02 (5-fold cross-validation) was achieved using the top 12 proteins from this list (Figure 3B).

The next objective was to develop a model predicting cognitive decline within the MCI group of 136 patients, 28 of whom showed progression during follow-up. A logistic regression-based classifier using a selected panel of 12 proteins achieved a ROC-AUC of just 0.64 ± 0.03 for distinguishing between the “progressed” and “stable” subgroups.

Therefore, a “universal” panel was constructed. To the 16 repeatedly L1-selected proteins distinguishing AD from Controls, 5 were added since they were consistently selected by L1-regularization for differentiating progressive MCI cases: C1S, TF, TTR, F12, and SERPINA7 (Figure 3C). To optimize the panel composition within the MCI subgroup, an iterative backward elimination approach was applied (see Section 4). The best ROC-AUC of 0.81 ± 0.1 (3-fold cross-validation) was achieved using the top 13 proteins (“universal panel”, Table 2). In addition, this panel yielded a ROC-AUC of 0.90 ± 0.02 for the AD vs. Control classifier (5-fold cross-validation, Figure 3B).

Notably, the protein panel outperformed the two standard clinical tests, MMSE and CDT (Figure 3D), whereas combining the protein markers with the clinical scores resulted in only a minor improvement of the ROC AUC to 0.82.

In addition, to assess the robustness of the AD vs. Control classifiers, we evaluated both panels using a cross-center training/testing scheme with the same model parameters. When trained on the MHRC dataset and tested on MH1, the ROC–AUC values were 0.94 for the Top-12 panel and 0.91 for the universal panel. Conversely, training on MH1 and testing on MHRC yielded ROC–AUC values of 0.90 and 0.80, respectively (Figures S1 and S2 in the Supplementary Materials). These differences likely reflect variations between the cohorts, which were mentioned in the Section 4.1.

Finally, the possibility of improving the classifier by including genetic characteristics was assessed. Among the tested genetic variants, the strongest associations were observed for APOE genotypes, which showed the expected opposite effects on circulating APOE levels: the ε2 allele was associated with higher (β = 0.37, *q* = 6.30 × 10^−3^), and the ε4 allele with lower APOE concentrations (β = −0.22, *q* = 6.30 × 10^−3^). Since the APOE level has already been considered in the developed classifier, we considered an additional link to the APOE polymorphism inappropriate. At the same time, none of the other loci analyzed retained significance after FDR correction, although some of them also showed nominal associations with plasma proteins (Supplementary Tables S5 and S6).

## 3. Discussion

Combining samples from different clinics into a common pool is often associated with some difficulties in aligning them according to specific characteristics. Importantly, both clinics that recruited participants for the current study generally used very similar approaches and criteria with minor differences in study methods, and the accuracy of the diagnoses is not in doubt.

Even more importantly, the analysis revealed that all the proteins showed either minor or inconsistent changes between cohorts. Average protein concentrations across diagnostic groups (Control, MCI, AD) were highly correlated between the two centers. This finding suggests that the datasets can be reliably merged for joint analysis, in line with common practice in large proteomic studies, where large cohorts are often built by integrating smaller datasets from different clinical centers or subcohorts. Such integration enhances statistical power while maintaining biological diversity.

The 43 proteins identified as significant (with *q*-value < 0.1) in the combined cohort represent several interconnected biological pathways relevant to AD, including lipoprotein particle remodeling and assembly, cholesterol efflux and reverse transport, acute-phase and inflammatory responses, and fibrinolysis and negative regulation of blood coagulation (Figure 3C,D). The major subgroup includes lipid transport and processing proteins (APOA1, APOA2, APOA4, APOB, APOC2, APOC3, APOE, APOM, PON1, PLTP, and LBP), which mediate cholesterol/phospholipid transport, antioxidant defense, and inflammation. Altered lipid metabolism and reduced antioxidant activity are well-recognized systemic hallmarks of AD, and *APOE*-ε4 remains the major genetic risk factor for the late-onset disease [33]. Another cluster includes coagulation and protease regulatory factors (FGA, FGB, FGG, F12, SERPINA1, SERPINA3, SERPINF2, HABP2, and C5), indicating links between coagulation, vascular inflammation, and complement activation. Complement and innate immune components (C1QB, C5, C9, CFB, CFI, HP, ORM1, and LRG1) underlie the systemic inflammatory activity that is characteristic of AD. The 43 list also includes transport and homeostatic proteins (TF, ALB, AHSG, PZP, and AFM) that are involved in iron and mineral metabolism, vitamin E transport, and maintaining systemic redox and inflammatory balance. Finally, SERPINF1, ATRN, LYZ, and GSN represent distinct functional categories encompassing neuroprotective and antiangiogenic signaling, cell adhesion and axonal guidance, antimicrobial defense, and cytoskeletal and inflammatory regulation. It is of particular importance that 37 of the 43 proteins are candidate biomarkers (CBs) of AD identified in other proteomic studies, and 19 of them were replicated in ≥3 independent cohorts (Table 1): APOE, APOA4, FGG, FGB, FGA, FN1, AFM, SERPINA3, TF, ORM1, SERPINA1, ALB, APOA1, HP, CFB, CFI, APOB, SERPINF1, and A1BG. Therefore, the findings from the functional analysis are appropriate to extend to well-reproducible markers whose significance was confirmed in this study.

Subdivision of the MCI group according to the degree of risk of progression to dementia based on the plasma proteomic profile is a particularly important task, which was also the focus of this work. Long-term follow-up of individual patients and the availability of their samples over time made it appropriate to evaluate the effectiveness of the binary AD vs. Control classifier in the MCI group, the subdivision of which required some refinement of the marker panel. Importantly, the 21 sorted proteins for building variations in classifiers included 12 overlaps with a set of important proteins from our previous study [31]: AFM, APOA4, ATRN, ORM1, PON1, SERPINF2, TF, APOE, PZP, C5, LBP, and FN1. Analysis of a wider number of samples and cohorts may provide further clarification. Overall, the results obtained here strongly suggest that further plasma studies using MRM may enable the development of a high-throughput proteomic panel for early assessment of Alzheimer’s disease risk.

## 4. Materials and Methods

### 4.1. Study Population

The study participants were recruited in two clinical centers (Moscow, Russia): the Mental Health Research Center (MHRC) and N.A. Alekseev Mental-Health Clinic No. 1 (MH1). The study was performed in accordance with the Declaration of Helsinki, and written informed consent was obtained from all participants. The study was approved by the MHRC (protocol No. 291, 18 July 2016) and MH1 (protocol No. 1, 25 January 2022) local ethical committees.

The MHRC participants included 46 patients with AD, 58 with MCI, and 51 Controls (Table 3). All patients were interviewed and underwent APOE and polygenic genotyping, and examined with the Mini-Mental State Examination (MMSE), clock drawing test (CDT), Boston naming test (BNT), Luria memory words test (LMWT), sound associations, and categorical associations subtests from the Mattis Dementia Rating Scale (MDRS) as described [31,34]. Other significant neurological diseases or psychiatric disorders were excluded. Control subjects had MMSE scores ≥ 28 (max 30), CDT scores ≥ 9 (max 10), BNT scores ≥ 51 (max 55), no history of neurological disease, no history or evidence of cognitive or functional decline, and no AD-specific MRI changes (Table 1). AD was diagnosed according to the criteria of the ICD-10 (International Classification of Diseases, 10th revision) as described [31]. The MCI group included patients diagnosed according to the criteria of the international consensus of the syndrome as described [31].

The MH1 participants (of ≥65 age old) included 49 patients with AD, 78 with MCI, and 49 cognitively unimpaired Controls. The participants were evaluated using a structured clinical interview, including MMSE, CDT, and MoCA scales; they answered a detailed questionnaire and participated in neurological examination and neuropsychological testing. Most of the participants underwent MRI brain screening and genotyping [34]. The patients were diagnosed according to the ICD-10 and have multiple impairments of cortical functions in at least two areas: memory and one of the cognitive functions. These included thinking (planning, programming, abstracting, and establishing cause-and-effect relationships), speaking, practicing, and gnosis. The age-matched volunteers for the Control group were recruited from patients who received periodic medical check-ups in the outpatient clinic No. 121 (Moscow). Psychiatric disorders, a positive family history of psychiatric disorders, substance abuse, or severe somatic diseases were excluded [34].

It is worth noting that some differences were observed between the two cohorts: (1) APOE(%e4+) and e3/e4 genotypes have no significant difference in the MH1-cohort between the Control and AD groups, while for MHRC, this difference was observed; (2) there is a significant (almost 2×) difference in e3/e3 between the Control and MCI cohort, while no difference was observed for the MHRC cohort. In our study, we focused on the combined cohort from both clinical centers to minimize these variations.

### 4.2. Quantitative Proteomic Analysis by LC-MRM-MS

Proteomic analysis of blood plasma samples (10 μL of sample) was performed using high-performance liquid chromatography coupled with mass spectrometry (LC-MS) in multiple reaction monitoring (MRM) mode. LC-MRM-MS analysis was implemented using an ExionLC™ UHPLC system (ThermoFisher Scientific, Waltham, MA, USA) coupled online to a SCIEX QTRAP 6500+ triple-quadrupole mass spectrometer (SCIEX, Concord, ON, Canada). For LC separation, a Zorbax Eclipse Plus reversed-phase (RP-UHPLC) column (2.1 × 150 mm, particle diameter 1.8 μm; Agilent, Santa Clara, CA, USA) was used. Peptides were separated at a flow rate of 0.4 mL/min for 60 min using a multistep gradient. The parameters for LC-MRM-MS analysis were adapted and optimized based on previous studies [31,35,36].

Quantitative analysis of selected plasma proteins was conducted using the BAK 125 kit (MRM Proteomics Inc., Montreal, QC, Canada) consisted of a set of stable isotope-labeled synthetic peptide standards (SIS), which were added to each digest sample as an internal standard for measuring the corresponding proteins. The protein concentrations (fmol per 1 µL of plasma) were determined via calibration curves generated using 1/(x × x)-weighted linear regression methods in Skyline Quantitative Analysis software (version 20.2.0.343, University of Washington) [37,38]. To ensure data quality, all protein and peptide measurements were validated according to ICH Bioanalytical Method Validation guidelines [39].

Protein concentration values were log_2_(C + 1)-transformed. The missing (NaN) values were imputed with the median values calculated within each diagnostic group (Control, MCI, AD). Batch effects were corrected using the neuroCombat algorithm [40], which applies an empirical Bayes framework to remove additive and multiplicative batch effects while preserving biological variation by including the diagnostic group (Control, MCI, AD) and continuous cognitive scores (MMSE and CDT) as covariates. The proteins identified in less than 70% of samples of any group were excluded from consideration (Supplementary Table S1).

### 4.3. Data Analysis

Statistical analyses and data visualization were carried out in Python (version 3.12.12) using the following packages: SciPy (version 1.16.3) [41], Seaborn (version 0.13.2) [42], Matplotlib (version 3.10.0) [43], and Pandas (version 2.2.2) [44]. Significant differences in protein concentrations in different groups were estimated using the Welch *t*-test. The false discovery rate (FDR) control Benjamini–Hochberg procedure was used to estimate type I error. The adjusted *p*-values were designated as *q*. Pearson’s coefficient was used to evaluate the correlation between features. STRING analysis was performed using the https://string-db.org resource. For statistical analysis, only unique patients were included, using the latest available blood sampling time point for those with repeated measurements.

To compare protein concentration levels between the two clinical centers, mean log_2_-transformed values were calculated for each diagnostic group (Control, MCI, AD) and averaged across groups. The comparability between protein means in the MH1 and MHRC cohorts was evaluated using linear regression (Python, scikit-learn package) [45].

Feature selection was performed using L1-regularized logistic regression with cross-validation (LogisticRegressionCV, scikit-learn, https://scikit-learn.org/stable/ (accessed on 14 October 2025) to identify stable protein markers (Supplementary Table S7). The model was repeatedly trained (1000 iterations) to estimate selection frequencies and average coefficients across runs.

The AD biomarker panel was optimized using a stepwise backward elimination approach starting from 16 proteins (L1-stable for AD/Control). At each step, one marker was removed, and model performance was re-evaluated using class-weighted logistic regression with L2 regularization and 5-fold cross-validation. The combination yielding the highest mean ROC-AUC was selected, resulting in a 12-protein “Top-12-for-AD panel”.

To construct the “universal panel”, the set of 16 L1-stable proteins identified for the AD/Control comparison was expanded by adding 5 additional proteins that were stably selected by L1-regularization in distinguishing MCI progressors from non-progressors (3-fold cross-validation, 1000 iterations) (Supplementary Table S8). The resulting 21-biomarker set was then refined using the same stepwise backward elimination procedure as applied for the Top-12-for-AD panel using 3-fold cross-validation.

A class-weighted logistic regression classifier was trained to discriminate AD vs. Control. The optimization used a nested cross-validation design: an inner 5-fold CV was applied to tune hyperparameters of the logistic regression model (solver, penalty, and regularization parameter), C = (10^−4^, 10^−2^), while an outer 5-fold CV was used to estimate model performance independently. The optimal decision threshold for calculating accuracy, sensitivity, and specificity was determined using Youden’s J statistic on the ROC curve.

For the Top-12-for-AD panel, the best model used liblinear + L2, C = 0.0464, achieving inner-CV AUC = 0.92 and outer-CV AUC = 0.93 ± 0.02, optimal threshold = 0.40, accuracy = 0.89, sensitivity = 0.96, and specificity = 0.82. For the “universal” panel, the best model also used liblinear + L2, C = 0.0464, yielding inner-CV AUC = 0.89 and outer-CV AUC = 0.90 ± 0.02, optimal threshold = 0.47, accuracy = 0.85, sensitivity = 0.87, and specificity = 0.83.

A class-weighted logistic-regression classifier was used to predict MCI progression (1) vs. non-progression (0). We evaluated three setups: (i) the universal protein panel only, (ii) the universal panel + MMSE + CDT, and (iii) MMSE + CDT alone. Model tuning used LogisticRegressionCV with L2 penalty and ‘lbfgs’ solver, optimizing C over a 10-point logarithmic grid from C = (10^−3^, 10^−2^), (class_weight = “balanced”, max_iter = 5000). Performance was estimated by a 3-fold stratified cross-validation.

Across all configurations, the logistic regression classifier (L2-regularized, lbfgs solver) demonstrated stable performance. The universal protein panel (13 proteins) achieved AUC = 0.81 ± 0.09 (optimal threshold = 0.57, accuracy = 0.85, sensitivity = 0.68, specificity = 0.89) with the best regularization parameter C = 0.0658, while the combined model with MMSE + CDT (15 features) reached AUC = 0.82 ± 0.10 (optimal threshold = 0.52, accuracy = 0.88, sensitivity = 0.75, specificity = 0.91) at C = 0.0081. In contrast, the MMSE + CDT alone performed worse (AUC = 0.62 ± 0.09, optimal threshold = 0.50, accuracy = 0.65, sensitivity = 0.64, specificity = 0.66, C = 0.001).

### 4.4. Polygenic Risk and Gene–Protein Correlation Analysis

Polygenic risk for AD was assessed in participants using a recently developed microarray-based assay that genotypes 21 pre-selected markers along with the ɛ alleles of the APOE gene [34]. The analyzed SNPs were rs6656401 (CR1 gene), rs6733839 (BIN1), rs35349669 (INPP5D), rs190982 (MEF2C), rs9271192 (HLA-DRB5/1), rs10948363—(CD2AP), rs271 8058 (NME8), rs1476679 (ZCWPW1), rs11771145 (EPHA1), rs28834970 (PTK2B), rs9331896 (CLU), rs10838725 (CELF1), rs7274581 (CASS4), rs983392 (MS4A6A), rs10792832 (PICALM), rs11218343 (SORL1), rs17125944 (FERMT2), rs10498633 (RIN-SLC24A), rs8093731 (DSG2), rs4147929 (ABCA7), and rs3865444 (CD33), in addition to APOE SNPs rs429358 and rs7412 [34].

Associations between genotypes and plasma protein concentrations were assessed using pairwise linear regression implemented in Python (SciPy package). Genetic variants were encoded numerically as wild type (WT) = 0, heterozygous (HET) = 1, and homozygous mutant (MUT) = 2. This additive coding scheme reflects the expected gene dosage effect and enables quantitative correlation and regression analyses between genotype and protein abundance (Supplementary Table S5).

Linear regression was performed using the linregress function, yielding the regression slope (β_1_), Pearson’s correlation coefficient (r), and two-sided *p*-value for the null hypothesis of no association (H_0_:β_1_ = 0). To account for multiple hypothesis testing, *p*-values were adjusted using the Benjamini–Hochberg false discovery rate (FDR) correction implemented in the multipletests function. The adjusted *p*-values were reported as *q*-values. The top-ranking gene–protein associations were selected based on FDR-adjusted significance levels (*q* < 0.1) (Supplementary Table S6).

## 5. Conclusions

A joint analysis of plasma samples from two clinical cohorts (MHRC and MH1, Moscow, Russia) confirmed a significant change in AD for 37 previously described candidate proteomic biomarkers, 19 of which had previously been shown to be reproducible in 3 or more independent cohorts. The set of 21 important proteins were selected using L1-regularized logistic regression and included ORM1, APOA4, LBP, HP, FN1, BCHE, APOE, PZP, A1BG, TF, SERPINA7, TTR, F12, C1S, AFM, CLU, C5, CP, ATRN, PON1, and SERPINF2. The universal panel included the first 13 proteins and showed ROC-AUC = 0.81 for distinguishing between stable and progressive patients with MCI, and ROC-AUC = 0.90 in the AD vs. Control comparison. Overlaps with our previous panel include ORM1, APOA4, LBP, FN1, APOE, PZP, AFM, ATRN, PON1, SERPINF2, and TF.

## Figures and Tables

**Table 1:** Significantly different proteins for the Controls vs. AD comparison in a joint analysis of MHRC and MH1 cohorts.

Protein Name	Gene Code	UniProt ID	Cohorts (n)	*q*-Value (FDR)
Afamin ^a^	AFM	P43652	4 [25,27]	1.75 × 10^−8^
**Apolipoprotein A-IV ^b^**	**APOA4**	**P06727**	6 [25,27]	1.77 × 10^−7^
Attractin	ATRN	O75882	2 [26,27]	1.10 × 10^−5^
Apolipoprotein A-I	APOA1	P02647	4 [25,27]	1.58 × 10^−5^
**Cholinesterase**	**BCHE**	**P06276**	-	1.58 × 10^−5^
**Serotransferrin**	**TF**	**P02787**	4 [25,26,27]	1.58 × 10^−5^
Alpha-2-antiplasmin	SERPINF2	P08697	-	2.00 × 10^−5^
Gelsolin	GSN	P06396	1 [26]	2.06 × 10^−5^
Serum paraoxonase/arylesterase 1	PON1	P27169	2 [26]	2.74 × 10^−5^
**Alpha-1-acid glycoprotein 1**	**ORM1**	**P02763**	3 [25,26]	2.74 × 10^−5^
**Haptoglobin**	**HP**	**P00738**	4 [25,26,27]	2.74 × 10^−5^
Alpha-1-antichymotrypsin	SERPINA3	P01011	4 [25,26]	2.81 × 10^−4^
Complement component C9	C9	P02748	1 [32]	2.93 × 10^−4^
Complement C1q subcomponent subunit B	C1QB	P02746	-	1.01 × 10^−3^
Apolipoprotein A-II	APOA2	P02652	1 [32]	1.67 × 10^−3^
Lysozyme C	LYZ	P61626	2 [26,32]	1.67 × 10^−3^
Fibrinogen beta chain	FGB	P02675	5 [25,26,27]	2.26 × 10^−3^
Serum albumin	ALB	P02768	5 [25,26,27]	2.26 × 10^−3^
Complement C5	C5	P01031	1 [26]	3.42 × 10^−3^
Fibrinogen gamma chain	FGG	P02679	6 [25,26,27]	3.55 × 10^−3^
Leucine-rich alpha-2-glycoprotein	LRG1	P02750	2 [26,32]	4.33 × 10^−3^
**Coagulation factor XII**	**F12**	**P00748**	1 [32]	4.67 × 10^−3^
Alpha-1-antitrypsin	SERPINA1	P01009	5 [25,26]	7.09 × 10^−3^
Pigment epithelium-derived factor	SERPINF1	P36955	3 [25,26,27]	1.14 × 10^−2^
**Apolipoprotein E**	**APOE**	**P02649**	6 [25,26,27]	1.63 × 10^−2^
Apolipoprotein B-100	APOB	P04114	3 [25,26,27]	1.63 × 10^−2^
Hyaluronan-binding protein 2	HABP2	Q14520	-	1.63 × 10^−2^
Alpha-2-HS-glycoprotein	AHSG	P02765	2 [27]	2.30 × 10^−2^
Apolipoprotein C-II	APOC2	P02655	1 [32]	2.57 × 10^−2^
Phospholipid transfer protein	PLTP	P55058	-	2.64 × 10^−2^
Kallistatin	SERPINA4	P29622	2 [32]	2.64 × 10^−2^
**Pregnancy zone protein**	**PZP**	**P20742**	2 [26]	2.98 × 10^−2^
Complement factor I	CFI	P05156	3 [25,26]	3.14 × 10^−2^
Complement factor B	CFB	P00751	4 [25,27]	3.96 × 10^−2^
**Alpha-1B-glycoprotein**	**A1BG**	**P04217**	3 [25,26,27]	4.05 × 10^−2^
Apolipoprotein C-III	APOC3	P02656	1 [32]	4.48 × 10^−2^
Fibrinogen alpha chain	FGA	P02671	4 [25,26,27]	4.58 × 10^−2^
**Fibronectin**	**FN1**	**P02751**	5 [25,26,27]	4.65 × 10^−2^
Apolipoprotein M	APOM	O95445	1 [32]	4.65 × 10^−2^
Vitamin K-dependent protein S	PROS1	P07225	1 [26]	4.65 × 10^−2^
Retinol-binding protein 4	RBP4	P02753	1 [27]	4.65 × 10^−2^
**Lipopolysaccharide-binding protein**	**LBP**	**P18428**	-	5.42 × 10^−2^
Cystatin-C	CST3	P01034	1 [26]	7.90 × 10^−2^

^a^ The gray background shows proteins that were important in a binary AD vs. control classification in the previous study performed with the MHRC cohort only; ^b^ the bold style indicates the proteins included in the universal panel.

**Table 2:** The universal panel proteins for assessment of the risk of progression to AD.

Protein Name	Gene Code	UniProt ID	*q*-Value (AD/Control)	Regulation in AD
Serotransferrin	TF	P02787	1.58 × 10^−5^	Down
Thyroxine-binding globulin	SERPINA7	P05543	9.31 × 10^−1^	-
Pregnancy zone protein	PZP	P20742	2.98 × 10^−2^	Down
Transthyretin	TTR	P02766	9.45 × 10^−1^	-
Cholinesterase	BCHE	P06276	1.58 × 10^−5^	Down
Coagulation factor XII	F12	P00748	4.67 × 10^−3^	Down
Apolipoprotein A-IV	APOA4	P06727	1.77 × 10^−7^	Down
Lipopolysaccharide-binding protein	LBP	P18428	5.42 × 10^−2^	Down
Alpha-1B-glycoprotein	A1BG	P04217	4.05 × 10^−2^	Up
Apolipoprotein E	APOE	P02649	1.63 × 10^−2^	Down
Alpha-1-acid glycoprotein 1	ORM1	P02763	2.74 × 10^−5^	Up
Fibronectin	FN1	P02751	4.65 × 10^−2^	Up
Haptoglobin	HP	P00738	2.74 × 10^−5^	Up

**Table 3:** Subject demographics.

	**Control**		**MCI**		**AD**	
	**MHRC**	**MH1**	**MHRC**	**MH1**	**MHRC**	**MH1**
N (samples)	51	49	58	78	46	49
Age (years)	66.8 ± 8.0	71.0 ± 6.2	71.7 ± 7.4	72.7 ± 7.1	73.5 ± 7.8	73.7 ± 7.1
Sex (%, F)	66.7	93.9	72.4	84.6	47.8	63.3
*APOE* (%, e4+)	9.5	40.0	18.8	20.0	43.5	42.8
e2/e2	0	0	0	0	0	2.0
e2/e3	11.9	26.7	12.5	7.7	4.3	6.1
e3/e3	78.6	33.3	68.7	72.3	52.2	49.1
e2/e4	2.4	6.7	0	1.5	2.2	0
e3/e4	7.1	33.3	18.8	16.9	15.2	36.7
e4/e4	0	0	0	1.5	26.1	6.1
MMSE	29.6 ± 0.7	28.4 ± 1.5	27.7 ± 2.5	27.1 ± 3.4	16.3 ± 5.9	8.1 ± 6.4
CDT	9.9 ± 0.27	8.7 ± 0.63	9.3 ± 1.16	6.2 ± 2.37	5.2 ± 2.69	1.4 ± 1.91
BNT	53.4 ± 1.6	Nm	49.0 ± 4.7	Nm	26.9 ± 17	Nm
МоСА	Nm	26.8 ± 1.0	Nm	22.6 ± 2.7	Nm	7.1 ± 4.6
Cardiovascular diseases (%)	69.7	82.3	67.2	78.2	73.9	93.9
Diabetes mellitus (%)	2.3	11.8	13.8	15.3	8.7	14.3
Gastrointestinal pathologies (%)	27.9	29.4	22.4	57.7	32.6	55.1
Genitourinary pathologies (%)	20.9	n.a.	37.9	n.a.	34.7	n.a.
Thyroid pathologies (%)	11.6	n.a.	12.1	n.a.	28.3	n.a.

Abbreviations: AD—Alzheimer’s disease; APOE—apolipoprotein E (genotype); BNT—Boston naming test; CDT—clock drawing test; MCI—mild cognitive impairment; MMSE—Mini-Mental State Examination; МоСА—Montreal Cognitive Assessment; MH1—the Mental-Health Clinic No. 1 cohort; MHRC—the Mental Health Research Center cohort; n.a.—not available; Nm—not measured. All average values are given ± SD.

## Data Availability

The original contributions presented in this study are included in the article/Supplementary Materials. Further inquiries can be directed to the corresponding authors.

## Supplementary Materials

The following supporting information can be downloaded at: https://www.mdpi.com/article/10.3390/ijms27010015/s1.

## Abbreviations

The following abbreviations are used in this manuscript:

AD	Alzheimer’s disease
AUC	Area under the ROC curve
Aβ	Amyloid-β
BNT	Boston naming test
CB	Candidate biomarker
CDT	Clock drawing test
CPTAC	Clinical Proteomic Tumor Analysis Consortium
CSF	Cerebrospinal fluid
MCI	Mild cognitive impairment
MMSE	Mini-mental state examination
MRM	Multiple reaction monitoring
MS	Mass spectrometry
p-tau	Phosphorylated tau
PEA	Proximity extension assay
ROC	Receiver operating characteristic
