# Supplementary material for: Plasma Protein Panel for Assessing the Risk of Alzheimer’s Disease by MRM-MS Analysis: The Study of Two Independent Clinical Cohorts
Source: Int J Mol Sci. 2025 Dec 19;27(1):15. doi: 10.3390/ijms27010015 (PMC12786224; doi:10.3390/ijms27010015)
Supplement: Supplementary file 1 [file ijms-27-00015-s001.zip › Figures S1-S2.docx]

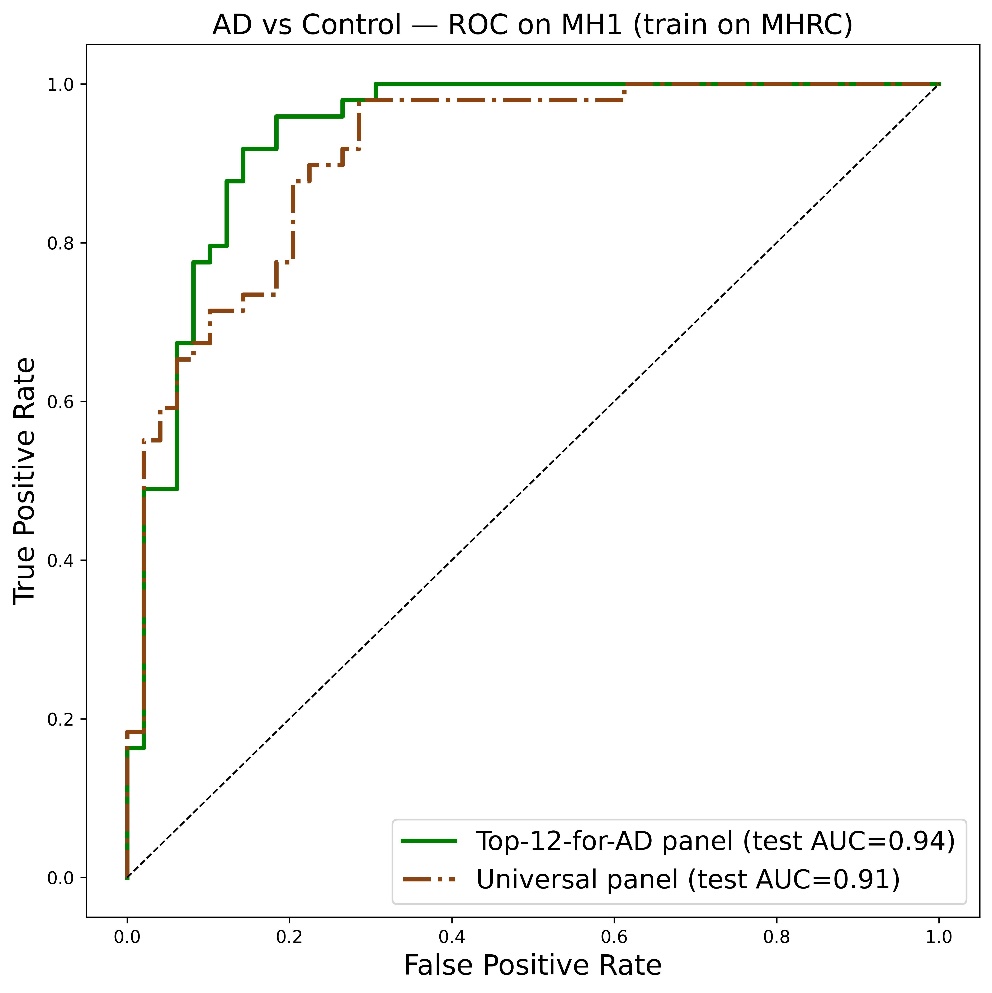


Figure S1. ROC curves for AD vs. Control classification trained on the MHRC cohort and tested on the MH1 cohort.


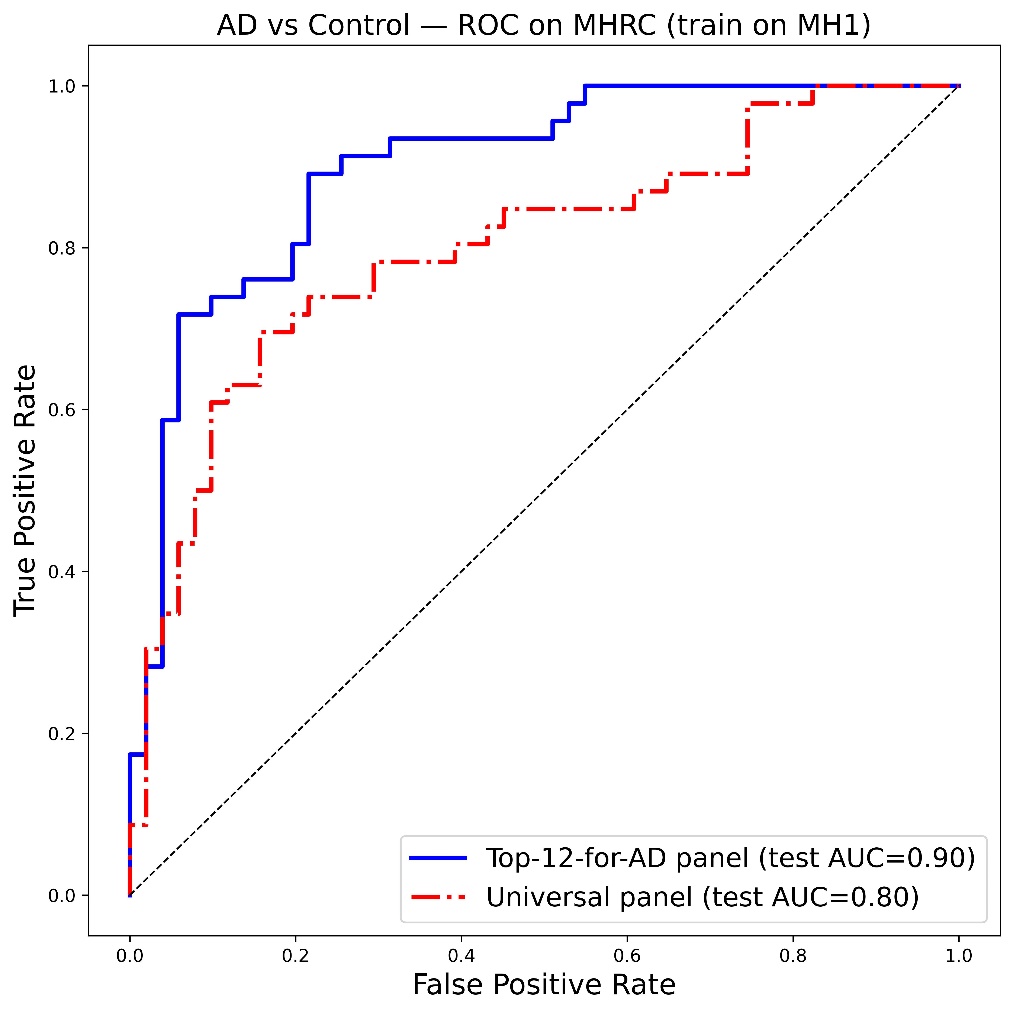


Figure S2. ROC curves for AD vs. Control classification trained on the MH1 cohort and tested on the MHRC cohort.
